# Colorectal Cancer Screening Amid COVID‐19 in Japan: Analysis From the 2021–2022 JACSIS Study

**DOI:** 10.1002/cam4.70859

**Published:** 2025-04-18

**Authors:** Hiroaki Saito, Aminu Kende Abubakar, Akihiko Ozaki, Daisuke Hori, Michio Murakami, Yudai Kaneda, Masaharu Tsubokura, Takahiro Tabuchi

**Affiliations:** ^1^ Department of Radiation Health Management Fukushima Medical University Fukushima Japan; ^2^ Department of Internal Medicine Soma Central Hospital Soma Japan; ^3^ Graduate School of Public Health, St. Luke's International University Chuo‐ku Japan; ^4^ Division of Population Data Science National Cancer Center Institute for Cancer Control Tokyo Japan; ^5^ Breast and Thyroid Center Jyoban Hospital of Tokiwa Foundation Iwaki Japan; ^6^ Occupational and Aerospace Psychiatry Group Institute of Medicine, University of Tsukuba Tsukuba Japan; ^7^ Center for Infectious Disease Education and Research The University of Osaka Suita Japan; ^8^ School of Medicine, Hokkaido University Sapporo Japan; ^9^ Division of Epidemiology School of Public Health, Tohoku University Graduate School of Medicine Sendai Japan

**Keywords:** colorectal cancer screening, COVID‐19, participation trends, socioeconomic status

## Abstract

**Background:**

Maintaining a high participation rate is crucial for effective colorectal cancer (CRC) screening. The COVID‐19 pandemic placed a significant burden on healthcare facilities, which hindered CRC screening efforts. However, the effects of the prolonged pandemic on CRC screening remain unclear.

**Methods:**

We analyzed data from the Japan COVID‐19 and Society Internet Survey in September 2021 and 2022 to examine CRC screening participation over the past year. We also evaluated the association between CRC screening participation in 2022 and the participation status, future screening intentions, background characteristics, and anxiety about COVID‐19 measured using the fear of coronavirus disease 2019 scale (FVC‐19S) from the 2021 survey..

**Results:**

Of the 13,261 respondents, 40.5% reported undergoing CRC screening in 2021, while 48.7% did so in 2022. Multivariable Poisson regression analysis showed that significant factors associated with CRC screening participation in 2022 included being male (adjusted incidence risk ratio [aIRR] 1.07, 95% confidence interval [CI]; 1.00–1.14, *p* = 0.026), age in the 40s (aIRR 0.89, 95% CI; 0.81–0.97, *p* = 0.012) and 50s (aIRR 0.89, 95% CI; 0.82–0.98, *p* = 0.011), being unmarried (aIRR 0.88, 95% CI; 0.82–0.95, *p* = 0.001), and employment status such as self‐employed (aIRR 0.86, 95% CI; 0.76–0.97, *p* = 0.012) or unemployed (aIRR 0.86, 95% CI; 0.81–0.92, *p* < 0.01). Having an FVC‐19S score below 21 was also a factor (aIRR 0.95, 95% CI; 0.90–1.00, *p* = 0.032).

**Conclusions:**

Although CRC screening rates increased from 2021 to 2022, a significant proportion of respondents still reported not undergoing screening, highlighting the importance of assessing the long‐term impact of COVID‐19 and identifying factors that make screening less accessible.

## Graphical abstract

Colorectal cancer (CRC) screening participation in Japan increased from 40.5% in 2021 to 48.7% in 2022, indicating partial recovery from pandemic disruptions. Key factors negatively affecting 2022 screening participation included younger age (40s–50s), being unmarried, employment status (self‐employed or unemployed), and lower COVID‐19 anxiety scores, highlighting persistent barriers to screening despite overall improvement.

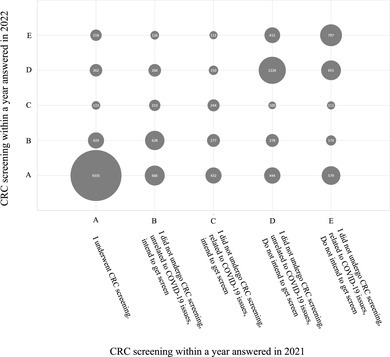

## 1 Introduction

Cancer screening plays a pivotal role in disease prevention and early detection, significantly contributing to reduced cancer mortality and the mitigation of overall life expectancy loss. The effectiveness of screenings for cancers such as colorectal, cervical, and breast cancer has been well established through numerous studies, prompting both nationwide and municipal implementation efforts [1, 2, 3]. Effective cancer screening necessitates a well‐managed system, tests with high sensitivity and specificity, and seamless integration with follow‐up treatment [4, 5]. Among these factors, the participation rate of the target population in screenings is a crucial indicator, prompting efforts to identify barriers and enhance participation rates [6]. However, such cancer screening initiatives are known to be vulnerable to large‐scale disasters and significant social changes. For example, following the Great East Japan Earthquake, the participation rates for colorectal and breast cancer screenings significantly declined in the most affected areas over several years [7, 8]. Understanding these declines and implementing appropriate countermeasures is vital.

The COVID‐19 pandemic, which emerged in early 2020, caused a global public health crisis. Besides the direct increase in infections and deaths due to the virus, the pandemic brought about significant public health challenges through social isolation measures and restricted access to healthcare facilities [9, 10, 11]. The impact on cancer care has been particularly concerning, with reports from the UK indicating a decrease in new diagnoses for most solid tumors in 2020. Specifically, the short‐term prognosis for colorectal cancer (CRC) worsened, highlighting the need for enhanced screening and diagnostic measures [12]. The continued decline in CRC screening rates remains a significant issue, with reports indicating that, as of 2022, screening rates at federally qualified health centers in the United States had not returned to pre‐pandemic levels [13]. Addressing this situation with effective measures is crucial.

In Japan, a nationwide state of emergency was declared from late April to early May 2020, restricting inter‐prefectural movement [14]. However, the actual surge in COVID‐19 cases occurred towards the end of 2020, with intermittent outbreaks continuing thereafter, leading the Japanese government to issue region‐specific states of emergency four times by September 2021. During this period, many healthcare facilities prioritized COVID‐19 responses, leading to interruptions or delays in cancer screenings in several municipalities. Residents were advised to avoid non‐essential outings and were reluctant to visit hospitals for fear of contracting COVID‐19, further suppressing healthcare‐seeking behaviors [15]. Consequently, there are concerns about an increase in advanced‐stage cancers detected through screenings since the pandemic began [16, 17]. In response to the prolonged pandemic, the government has urged residents to continue necessary medical visits and cancer screenings, despite the ongoing crisis. Given this context, it is presumed that public perception of cancer screenings has evolved throughout the pandemic, but detailed analyses of the changes in screening behaviors in Japan are limited.

The aim of this study was to identify factors related to non‐participation in CRC during the pandemic, focusing on changes in CRC screening behaviors in 2020 and 2021.

## 2 Method

### 2.1 Setting and Participants

**FIGURE 1:**
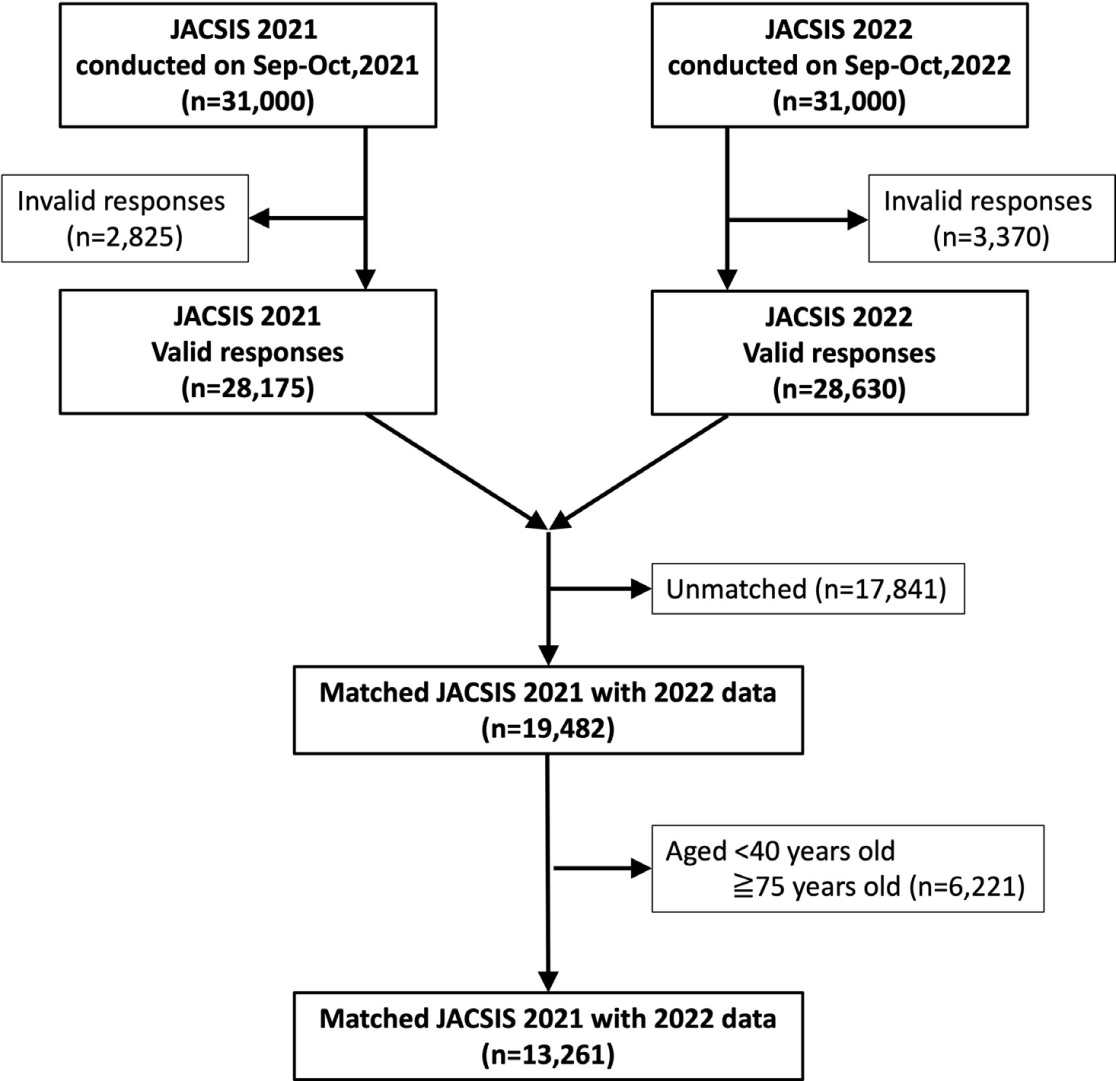
Flow chart of eligible participants for the study.

We utilized data from the Japan COVID‐19 and Society Internet Survey (JACSIS) study. JACSIS is a large‐scale annual internet survey conducted during the pandemic, capturing changes in health behaviors and identifying relevant factors, designed to estimate nationwide representative values [18, 19]. The survey is administered by an internet survey company (Rakuten insight marketing Inc., Tokyo, Japan) with approximately 2.2 million panelists and conducted online from September to October each year. In these surveys, potential participants were selected based on stratification by gender, age, and their prefecture of residence to ensure a sample that is as representative of the national population as possible. Participants are typically re‐invited from year to year, ensuring consistency in the survey cohort. Respondents were compensated with a small incentive for completing each survey. In this study, we focused on respondents who participated in both the surveys conducted from September to October 2021 and from September to October 2022. Out of the 31,000 respondents in 2021 and 32,000 respondents in 2022, we excluded invalid responses and created a panel data set of 19,482 individuals who participated in both surveys (Figure 1). Of these, we analyzed 13,261 individuals aged 40 to 74, who were the target for colorectal cancer screening in 2021 (Figure 1).

### 2.2 Variables

The primary outcome variable was participation in CRC screening within the past year, as asked in the 2022 survey. In Japan, annual fecal immunochemical testing for CRC is recommended for individuals aged 40 and above [20]. Those eligible for screening can undergo CRC screening either through public CRC screening provided by the city or through screening opportunities provided by the company they work for. Therefore, CRC screening is optimal for assessing annual cancer screening trends across genders. The questionnaire assessed whether respondents had undergone CRC screening in the past year (e.g., fecal occult blood test) using closed‐ended questions. While the fecal occult blood test (FOBT) was mentioned as an example, the inclusion of “etc.” was intended to encompass any type of fecal‐based CRC screening, including the fecal immunochemical test (FIT), which is increasingly used in Japan. Other screening procedures, such as colonoscopy, were not covered under this measure. Participants selected from predefined options indicating whether they had been screened, the screening outcome, and, if they had not participated, whether the reason was related to the COVID‐19 pandemic and their future screening intentions. COVID‐19‐related reasons included any pandemic‐associated factors, not limited to direct infection. For analysis, CRC screening participation was coded as a binary variable (1 = attended, 0 = did not attend). Responses indicating that screening had taken place (regardless of the outcome or if results were pending) were coded as attended, while responses indicating non‐attendance were coded as not attended. Similarly, pre‐pandemic screening patterns were categorized into regular, irregular, or no participation. The 2021 survey captured screening experiences from 2020 to the time of the survey, while the 2022 survey covered experiences from 2021 to the time of that survey.

Based on the 2021 survey responses, the following variables were included: age categories (40s, 50s, 60s, and 70s), marital status (categorized as married, never married, separated, and divorced), living arrangements (either living alone or with others), educational level as per ISCED (international standard classification of education, categorized into university degree or higher, junior high/high school, and vocational/junior/technical college), household annual income (classified as below 3 million Yen and 3 million Yen or above), and employment status (classified as employer, self‐employed, employee, and unemployed). The 3 million Yen threshold was used to define relative poverty for household income.

Health‐related variables included comorbidities (whether the respondent was receiving treatment for hypertension, diabetes, asthma, coronary artery disease, stroke, COPD, malignancy, or mental illness), smoking status (never used, former user, and current smoking), and drinking status (never, ever, and current). Additionally, we evaluated COVID‐19‐related anxiety using the Fear of COVID‐19 scale (FVC‐19s), classifying scores below 21 as low and scores of 21 or above as high, as defined in previous studies [21]. We also determined whether respondents lived in regions where a state of emergency was declared in 2021, based on their reported prefecture‐level residence.

### 2.3 Data Analysis

Firstly, descriptive analyses were conducted to examine participation in colorectal cancer screening in 2021 and 2022. Specifically, we described the results and reasons for participation or non‐participation in colorectal cancer screening within the past year as reported in 2021, their subsequent screening intentions, and their screening status in 2022. We used a chi‐square test to compare whether the proportion of individuals who underwent colorectal cancer screening in 2022 differed between those who did not undergo screening in 2021 for COVID‐19‐related reasons and those who did not undergo screening for non‐COVID‐19‐related reasons. Secondly, we performed multivariable Poisson regression analysis using all variables to identify factors associated with colorectal cancer screening participation in 2022. We verified multicollinearity among independent variables using the Variance Inflation Factor (VIF). Thirdly, we conducted a stratified analysis by age group (40s, 50s, 60s, and 70s) to assess variations in the association between employment status and colorectal cancer screening participation. Finally, we conducted stratified analyses based on the responses of the participants regarding CRC screening results and subsequent intentions reported in 2021. All statistical analyses were performed using Stata version 15 (StataCorp LP, College Station, TX).

### 2.4 Ethical Approval

This study complied with the principles set forth in the Declaration of Helsinki. The research protocols for the JACSIS study were approved by the Research Ethics Committee of the Osaka International Cancer Institute (Approval No. 20084‐2). All participants provided informed consent at the time of their registration with Rakuten Insight, which administered the survey. Before completing the online questionnaire, participants received an informed consent form and were informed that they could withdraw from the study at any point.

## 3 Result

### 3.1 Background of Participants

The background characteristics of the participants are shown in Table 1. There were 6726 women (50.7%), and the average age was 57.2 years (SD 10.2). A total of 4239 participants (32.0%) had pre‐existing conditions and were receiving regular medical care. Additionally, 10,169 participants (76.7%) resided in areas where a state of emergency was declared in 2021. Based on the questionnaire items, the Fear of COVID‐19 score was 21 or higher for 4682 participants (35.3%). Regarding colorectal cancer screening before COVID‐19, 5033 participants (38%) reported undergoing annual screening, 2604 (19.6%) reported irregular screening, and 5624 (42.4%) reported never having participated.

**TABLE 1:** Participant characteristics in 2021 (JACSIS study).

Variables	Number of participants
Total	13,261 (100)
Male	6535 (49.3)
Female	6726 (50.7)
Age	
40s	3892 (29.4)
50s	3535 (26.7)
60s	3815 (28.8)
70s	2019 (15.2)
Marital status	
Married	9137 (68.9)
Unmarried	2510 (18.9)
Widowed	459 (3.5)
Divorced	1155 (8.7)
Housing situation	
Living alone	2443 (18.4)
Living with others	10,818 (81.6)
Highest education	
Middle/High School	3959 (29.9)
Vocational School/Junior College/Technical College	3268 (24.6)
University and above	6034 (45.5)
Employment status	
Employer	473 (3.6)
Self‐employed	746 (5.6)
Employee	7050 (53.2)
Unemployed	4992 (37.6)
Annual household income	
3 million Yen or less	2306 (17.4)
Greater than 3 million Yen	8141 (61.4)
Unanswered	2814 (21.2)
Smoking status	
No smoking history	7446 (56.1)
Former‐user	3119 (23.5)
Smoking (paper)	1450 (10.9)
Smoking (electronic)	638 (4.8)
Smoking (both)	608 (4.6)
Drinking status	
Never	4977 (37.5)
Ever	902 (6.8)
Current	7382 (55.7)
Comorbidities	
Present	4239 (32.0)
Absent	9022 (68.0)
State of emergency in 2021 for the place of residence	
Issued	10,169 (76.7)
Not issued	3092 (23.3)
Fear of Covid‐19 scale score	
7–15	3520 (26.5)
16–20	5059 (38.1)
21–25	3791 (28.6)
26–35	891 (6.7)
Participation in colorectal cancer screening before COVID‐19	
Regular screening participation	5033 (38.0)
Irregular screening participation	2604 (19.6)
Never participation	5624 (42.4)

### 3.2 Participation Rates in 2021 and 2022

**FIGURE 2:**
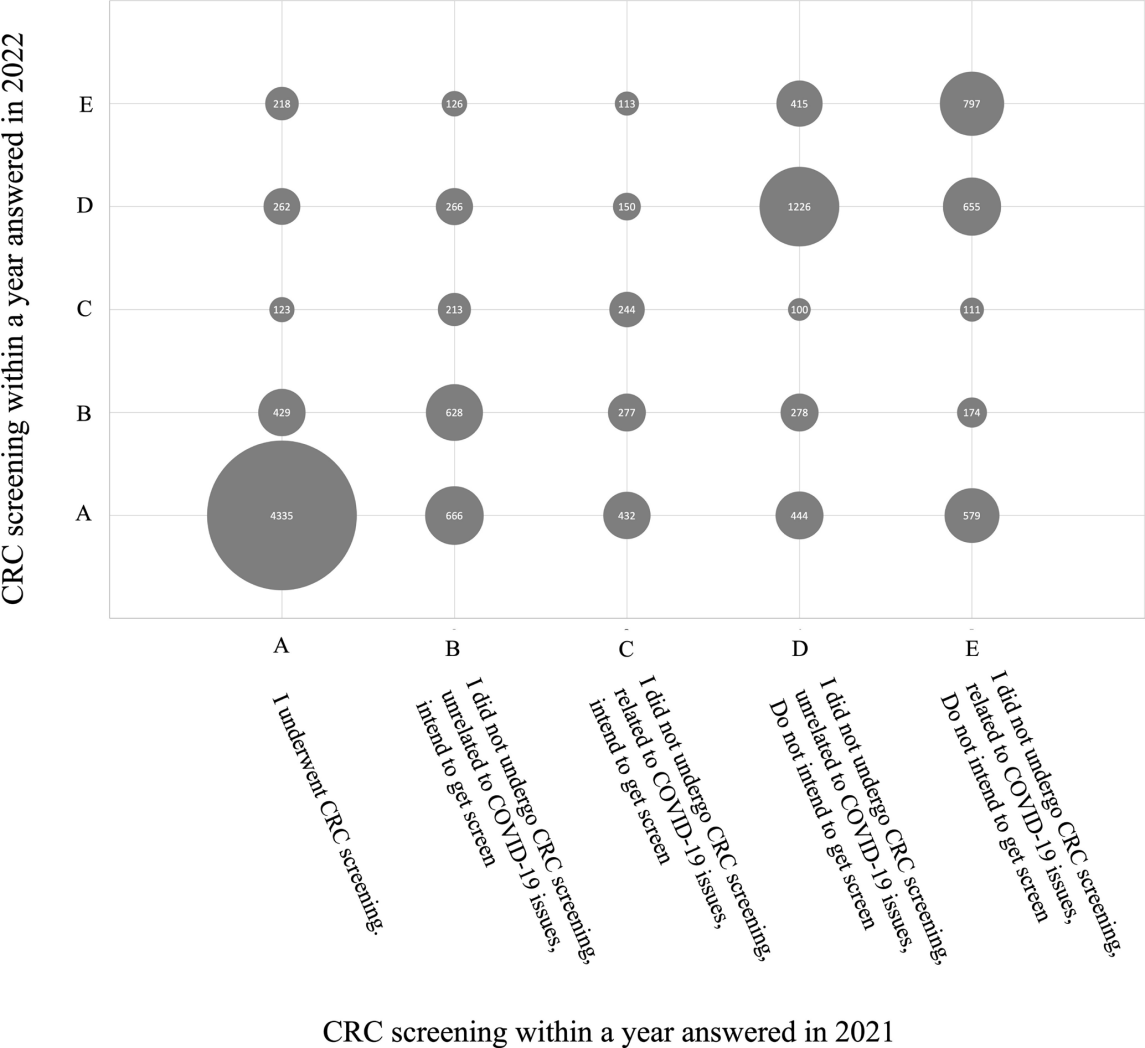
Bubble chart of matrix of responses to 2‐year colorectal cancer screening participation. The size of the bubble reflects the number of people, and the actual number is listed in the bubble.

In 2021, 5367 participants (40.5%) reported undergoing colorectal cancer screening. Among the 7894 participants (59.5%) who did not undergo screening, 1899 (14.3%) reported inability to screen due to reasons unrelated to COVID‐19, yet intended future participation, while 1216 (9.2%) cited COVID‐19‐related reasons but planned to undergo screening later, 2463 (18.6%) could not screen for reasons unrelated to COVID‐19 and did not intend to screen in the future, and 2316 (17.5%) cited COVID‐19‐related reasons and did not intend to screen in the future. In 2022, 6456 participants (48.7%) reported undergoing colorectal cancer screening. Among the 6805 participants (51.3%) who did not screen, 1786 (13.5%) cited reasons unrelated to COVID‐19 but intended to screen in the future, 791 (6.0%) cited COVID‐19‐related reasons but intended to screen in the future, 2559 cited reasons unrelated to COVID‐19 and did not intend to screen in the future, and 1669 (12.6%) cited COVID‐19‐related reasons and did not intend to screen in the future. Figure 2 illustrates the matrix of these responses. Of those screened in 2021, 4335 participants (80.8%) also underwent screening in 2022 (refer to Figure 2, column A). Of the 3115 participants who did not screen in 2021 but intended to (columns B and C of Figure 2), 1098 (35.2%) screened in 2022. Conversely, of the 4779 participants who did not screen in 2021 and did not intend to screen thereafter (columns D and E of Figure 2), 1023 (21.4%) screened in 2022. Those who cited COVID‐19‐related reasons for not screening and not intending to screen thereafter were more likely to screen in 2022 compared to those citing non‐COVID‐19‐related reasons (18.0% vs. 25.0%, *p* < 0.05). Table S1 shows the screening rates in 2022 by various attributes.

### 3.3 Factors Associated With Colorectal Cancer Screening in 2022

Table 2 presents the results of the multivariable Poisson regression analysis identifying factors associated with colorectal cancer screening in 2022. Significant factors for CRC participation in 2022 included gender (male: Incidence Risk Ratio (IRR) 1.07, 95% confidence interval (95% CI) 1.01–1.14, *p* = 0.026), age (40s: IRR 0.89, 95% CI 0.81–0.97, *p* = 0.012; 50s: IRR 0.89, 95% CI 0.82–0.98, *p* = 0.011; reference = 70s), marital status (never married: IRR 0.88, 95% CI 0.82–0.95, *p* = 0.001, reference = married), employment status (self‐employed: IRR 0.86, 95% CI 0.76–0.97, *p* = 0.012; unemployed: IRR 0.86, 95% CI 0.81–0.92, *p* < 0.001; reference = employee), and FVC‐19S of 21 or less (IRR 0.95, 95% CI 0.90–1.00, *p* = 0.032). In addition, those who did not participate in 2021 compared to those who did in 2021 were associated with lower participation rates in the CRC in 2022 (could not screen for non‐COVID‐19 reasons but intended to: IRR 0.45, 95% CI 0.41–0.49, *p* < 0.001; could not screen for COVID‐19‐related reasons but intended to: IRR 0.45, 95% CI 0.41–0.50, *p* < 0.001; could not screen for non‐COVID‐19 reasons and did not intend to: IRR 0.23, 95% CI 0.21–0.26, *p* < 0.001; could not screen for COVID‐19‐related reasons and did not intend to: IRR 0.32, 95% CI 0.30–0.35, *p* < 0.001). Poisson regression analysis stratified by age group, using the same variables, showed that being unemployed was associated with a statistically significant lower likelihood of participating in screening—particularly among individuals in their 40s (IRR 0.75, 95% CI 0.64–0.88, *p* < 0.001), 50s (IRR 0.81, 95% CI 0.71–0.94, *p* < 0.001), and 60s (IRR 0.89, 95% CI 0.81–0.99, *p* = 0.02)—but not among those in their 70s (IRR 0.99, 95% CI 0.84–1.17, *p* = 0.89).

**TABLE 2:** Multivariable regression analysis of colorectal cancer screening participation in 2022.

Variables	aIRR (95% CI)	*p*
Gender		
Female	Reference	
Male	1.07 (1.01–1.14)	0.026
Age		
70s	Reference	
40s	0.89 (0.81–0.97)	0.012
50s	0.89 (0.82–0.98)	0.011
60s	0.97 (0.89–1.04)	0.366
Marital status		
Married	Reference	
Never married	0.88 (0.82–0.95)	0.001
Widowed	1.04 (0.91–1.20)	0.552
Divorced	0.99 (0.90–1.08)	0.744
Highest education		
University and above	Reference	
Middle/High School	0.97 (0.91–1.03)	0.317
Vocational School/Junior College/Technical College	0.95 (0.89–1.01)	0.121
Employment type		
Employee	Reference	
Employer	1.00 (0.88–1.14)	0.982
Self‐employed	0.86 (0.76–0.97)	0.012
Unemployed	0.86 (0.81–0.92)	< 0.001
Annual household income		
Greater than 3 million Yen	Reference	
3 million Yen or less	0.97 (0.89–1.04)	0.357
No response	0.97 (0.91–1.03)	0.283
State of emergency in 2021 for the place of residence		
Not issued	Reference	
Issued	1.00 (0.94–1.06)	0.927
Fear of Covid‐19 scale score		
Less than 21	0.95 (0.90–1.00)	0.032
21 and greater	Reference	
Smoking		
Never‐user	Reference	
Former‐user	0.97 (0.91–1.03)	0.322
Current smoking (paper)	0.93 (0.85–1.02)	0.123
Current smoking (electronic)	0.99 (0.88–1.12)	0.898
Current smoking (both)	0.93 (0.82–1.05)	0.256
Alcohol use		
Never	Reference	
Ever	0.93 (0.83–1.04)	0.186
Current	1.01 (0.95–1.06)	0.816
Comorbidities		
Present	Reference	
Absent	0.97 (0.92–1.03)	0.315
Colorectal cancer screening in 2021		
Participated in 2021	Reference	
Other reason willing to participate	0.45 (0.41–0.49)	< 0.001
COVID‐19 related issue willing to participate	0.45 (0.41–0.50)	< 0.001
Other reason unwilling to participate	0.23 (0.21–0.26)	< 0.001
COVID‐19 related issue unwilling to participate	0.32 (0.30–0.35)	< 0.001

Abbreviation: aIRR, adjusted incidence risk ratio.

Table 3 shows the factors associated with colorectal cancer screening participation in 2022, stratified by whether the participants underwent screening in 2021. Among those who attended CRC screening in 2021, being in their 40s was negatively associated with participation in CRC screening in 2022 (IRR 0.88, 95% CI 0.79–0.98, *p* = 0.021; reference = 70s). Conversely, among those who did not attend in 2021, male (IRR 1.33, 95% CI 1.20–1.48, *p* < 0.001), FVC‐19 score of 21 or higher (IRR 1.22, 95% CI 1.11–1.33, *p* < 0.001), citing COVID‐19‐related reasons for not screening in 2021 (IRR 1.16, 95% CI 1.07–1.27, *p* = 0.001), and having an intention to screen (IRR 1.66, 95% CI 1.52–1.81, *p* < 0.001), are positively associated with participation in CRC screening in 2022. On the other hand, never married status (IRR 0.78, 95% CI 0.69–0.88, *p* < 0.001; reference = married), self‐employed (IRR 0.62, 95% CI 0.51–0.76, *p* < 0.001; reference = employee), unemployed (IRR 0.69, 95% CI 0.61–0.77, *p* < 0.001; reference = employee), and without comorbidities (IRR 0.91, 95% CI 0.83–1.00, *p* = 0.048) are negatively associated with participation in CRC screening in 2022. Table S2 shows the results of further stratified analyses among those who did not screen in 2021, with overall trends remaining consistent.

**TABLE 3:** Factors related to colorectal cancer screening participation in 2022, stratified by whether the participants underwent the screening in 2021.

	Attendee on CRC screening in 2021		Non‐attendee on CRC screening in 2021	
	aIRR (95% CI)	*p*	aIRR (95% CI)	*p*
Gender				
Female	Reference		Reference	
Male	0.98 (0.91–1.05)	0.529	1.33 (1.20–1.48)	< 0.001
Age				
70s	Reference		Reference	
40s	0.88 (0.79–0.98)	0.021	0.93 (0.80–1.10)	0.399
50s	0.93 (0.84–1.04)	0.187	0.87 (0.74–1.02)	0.076
60s	0.96 (0.88–1.05)	0.369	1.02 (0.88–1.18)	0.797
Marital status				
Married	Reference		Reference	
Never married	0.95 (0.87–1.04)	0.268	0.78 (0.69–0.88)	< 0.001
Widowed	1.06 (0.90–1.25)	0.481	0.98 (0.76–1.27)	0.901
Divorced	0.95 (0.85–1.07)	0.383	1.04 (0.89–1.21)	0.661
Highest education				
University and above	Reference		Reference	
Middle/High School	0.97 (0.90–1.05)	0.481	0.97 (0.88–1.08)	0.630
Vocational School/Junior College/Technical College	0.95 (0.87–1.03)	0.186	0.96 (0.85–1.08)	0.461
Employment type				
Employee	Reference		Reference	
Employer	1.02 (0.86–1.20)	0.846	0.95 (0.77–1.17)	0.623
Self‐employed	1.05 (0.90–1.22)	0.543	0.62 (0.51–0.76)	< 0.001
Unemployed	0.98 (0.90–1.06)	0.538	0.69 (0.61–0.77)	< 0.001
Annual household income				
Greater than 3 million Yen	Reference		Reference	
3 million or less	1.02 (0.93–1.12)	0.732	0.88 (0.78–1.00)	0.057
No response	0.99 (0.91–1.07)	0.717	0.94 (0.84–1.05)	0.252
State of emergency in 2021 for the place of residence				
Not issued	Reference		Reference	
Issued	1.00 (0.93–1.07)	0.965	1.00 (0.9–1.11)	0.992
Fear of Covid‐19 scale score				
Less than 21	Reference		Reference	
21 and greater	0.98 (0.92–1.05)	0.546	1.22 (1.11–1.33)	< 0.001
Smoking				
Never‐user	Reference		Reference	
Former‐user	0.97 (0.90–1.05)	0.466	0.97 (0.86–1.08)	0.555
Current smoking (paper)	0.97 (0.87–1.08)	0.576	0.87 (0.75–1.00)	0.051
Current smoking (electronic)	0.98 (0.84–1.14)	0.821	0.99 (0.82–1.21)	0.954
Current smoking (both)	0.90 (0.77–1.06)	0.220	0.95 (0.78–1.15)	0.593
Alcohol use				
Never	Reference		Reference	
Ever	0.93 (0.81–1.07)	0.326	0.93 (0.78–1.10)	0.390
Current	1.01 (0.95–1.08)	0.709	1.02 (0.92–1.12)	0.751
Comorbidities				
Present	Reference		Reference	
Absent	1.01 (0.95–1.08)	0.755	0.91 (0.83–1.00)	0.048
Reason of non‐participation				
Other reason	—		Reference	
COVID‐19 related	—		1.16 (1.07–1.27)	0.001
Intention to screening				
Unwilling	—		Reference	
Willing	—		1.66 (1.52–1.81)	< 0.001

Abbreviation: aIRR, adjusted incidence risk ratio.

## 4 Discussion

This study conducted a detailed analysis of involvement in CRC screening during the COVID‐19 pandemic through a large‐scale, two‐year survey. Utilizing panel data, we examined trends in CRC screening uptake and found that the overall participation rate increased from 2021 to 2022. Interestingly, the inability to undergo screening in 2021 due to COVID‐19 appeared to motivate participation in 2022, suggesting a reduction in the pandemic's impact on CRC screening. However, the majority of individuals who did not undergo screening in 2021 also refrained from participating in 2022, indicating that screening behaviors tend to become entrenched. These findings highlight the impact of COVID‐19 on CRC screening and emphasize the need for targeted interventions for populations that remain unscreened.

This study suggests that the impact of COVID‐19 on CRC screening lessened from 2021 to 2022. In this survey, 38% of respondents reported participating in CRC screening regularly and 19.6% irregularly before COVID‐19. However, in 2021, 40.5% and in 2022, 48.7% reported actually attending the screenings. Similarly, a nationally representative survey conducted by the Ministry of Health, Labour and Welfare shows that colorectal cancer screening uptake in Japan slightly increased from 47.8% to 49.1% among males and from 40.9% to 42.8% among females between 2019 and 2022 [22]. While this reflects a modest national recovery, the more pronounced increase observed in our study suggests that certain populations may have experienced a more substantial rebound in screening participation. It is noteworthy that a significant number of individuals reported being unable to undergo CRC screening in 2022 due to the pandemic. Many countries have reported a decrease in CRC screening participation rates during the peak of the COVID‐19 pandemic, noting declines especially in early 2020, followed by subsequent recoveries. For example, an Italian study noted a 5.1% decrease in participation rates during the peak period from January to March 2020, but rates returned to 2019 levels once screening activities resumed [23]. Similarly, in the United States, the number of fecal occult blood tests performed returned to 2019 levels by September 2020 [24]. Conversely, long‐term impacts on screening trends have also been reported. In Canada, CRC screening participation in 2022 was still down by 4% compared to pre‐pandemic levels [25]. These differences in impact are likely due to variations in the COVID‐19 pandemic's status and the corresponding healthcare policies in each country. Our study also shows that while CRC screening rates in Japan are on a recovery trend, the impact of the pandemic persisted into 2022, highlighting the need for ongoing interventions. Notably, 65% of individuals who intended to undergo CRC screening in 2021 but did not, also did not undergo screening in 2022. Identifying the barriers preventing these groups from participating in screening is crucial for improving screening rates.

This study's finding that social factors such as age and work environment are associated with adherence to fecal occult blood screening aligns with previous research. Generally, it is known that individuals with lower educational levels, poverty, poor employment conditions, and younger age are less likely to participate in CRC screening [26]. In Japan, CRC screenings are often provided at workplaces alongside public health screenings, making the influence of occupation and income particularly pronounced during the COVID‐19 pandemic. In fact, when analyzed by age group in the current study, the negative association of unemployment with screening participation was more pronounced among those in their 40s–60s, the working age group. Furthermore, the finding that men were more likely to undergo CRC screening in this study contrasts with previous reports suggesting that women are more likely to participate in the screenings [27]. Reports in Japan have also indicated that men are less likely to participate in CRC screenings compared to women [7]. On the other hand, our findings are consistent with trends observed in the Ministry of Health, Labour and Welfare's national health survey, which reports higher screening rates among men for stomach, lung, and colorectal cancer [22], while the survey provides only descriptive data without statistical analysis. Further research is needed to investigate the factors influencing gender differences in participation across these screening programs. In addition, it remains unclear whether the impact of socioeconomic status on CRC screening has changed before and after the pandemic. Globally, studies have reported both an increase in disparities due to COVID‐19 and a narrowing of pre‐existing gaps [28]. This study did not consider the impact of job losses or changes in work patterns, including remote work, during the pandemic, representing a limitation that future research should address. Analyzing changes in the influence of social factors on CRC screening participation in Japan during the pandemic is crucial for developing strategies to improve screening rates.

This study reveals that prior participation in screenings and the intention to participate in future screenings are strongly associated with subsequent screening participation. It may also highlight the fixed nature of groups who either consistently undergo CRC screening or consistently do not, even during the COVID‐19 pandemic. These findings align with previous research indicating that knowledge about CRC and screenings, as well as a lack of trust in healthcare providers, can significantly impact an individual's willingness to undergo screenings, potentially affecting lifetime adherence to CRC screening [29]. The Japanese government and the Ministry of Health, Labour and Welfare have set a target CRC screening rate of 80%. To achieve this goal, it is crucial to focus on engaging those who consistently do not participate in CRC screenings. Analyzing trends in CRC screening participation and the factors influencing it, and then implementing personalized messaging to encourage participation [30], may be an effective approach.

This study has several limitations. First, since the survey collected information on screening status for the year prior to the response, the impact of the COVID‐19 pandemic during its peak from March to May 2020 may be underestimated. Second, as the study focused on trends in fecal occult blood testing, it may not have adequately adjusted for the influence of other screening methods, such as past colonoscopies. However, by collecting information on respondents' medical histories and healthcare visits, the analysis attempts to account for these effects. Third, the reasons for not undergoing CRC screening related to COVID‐19 include subjective elements and, finally, the survey is online self‐reported; there are potentials for response bias and inaccuracy, and results may not be representative of the broader population. Fourth, the sample may not fully represent the general population, as most respondents were educated beyond high school, earned over 3 million Yen, and were internet users due to the online survey format. These factors may influence health behaviors and limit the generalizability of the findings. Nonetheless, the aim of this study is to understand how participants perceive these reasons in relation to their screening behavior.

## 5 Conclusion

A two‐year survey revealed an increasing trend in CRC screening rates from 2021 to 2022. However, even in 2022, many respondents cited the COVID‐19 pandemic as a reason for not participating in CRC screening, indicating concerns about its long‐term impact. This study highlights the persistent segmentation of groups regarding CRC screening participation. Identifying the factors contributing to this fixed pattern and devising appropriate interventions are crucial for selecting suitable target groups for intervention.

## Author Contributions

**Hiroaki Saito:** conceptualization (equal), data curation (equal), formal analysis (equal), methodology (equal), writing – original draft (equal). **Aminu Kende Abubakar:** formal analysis (equal), writing – original draft (equal). **Akihiko Ozaki:** conceptualization (equal), writing – review and editing (equal). **Daisuke Hori:** validation (supporting), writing – original draft (supporting), writing – review and editing (supporting). **Michio Murakami:** methodology (equal), supervision (equal), writing – review and editing (equal). **Yudai Kaneda:** writing – review and editing (equal). **Masaharu Tsubokura:** writing – review and editing (equal). **Takahiro Tabuchi:** funding acquisition (equal), project administration (equal), resources (equal), supervision (equal), writing – review and editing (equal).

## Ethics Statement

Ethical approval to conduct the present study was obtained from the ethics committee of the Osaka International Cancer Institute (approval number: 20084‐6). All procedures were performed in accordance with the ethical standards of the institutional and/or national research committees and the Declaration of Helsinki and its later amendments, or comparable ethical standards.

## Consent

All participants provided web‐based informed consent at registration.

## Conflicts of Interest

A.O. receives personal fees from MNES Inc. and Kyowa Kirin Inc. A.O. and H.S. received personal fees from Taiho Pharmaceutical Co. Ltd. outside the submitted work. The other authors declare no conflicts of interest.

## Supporting information


**Table S1.** The colorectal cancer screening participation rates in 2022 by the characteristics.
**Table S2.** Factors related to colorectal cancer screening participation in 2022, among those who did not screen in 2021.

## Data Availability

The data that support the findings of this study are available from the corresponding author upon reasonable request.
