# Supplementary material for: Colorectal Cancer Screening Amid COVID‐19 in Japan: Analysis From the 2021–2022 JACSIS Study
Source: Cancer Med. 2025 Apr 18;14(8):e70859. doi: 10.1002/cam4.70859 (PMC12007422; doi:10.1002/cam4.70859)
Supplement: Supplementary file 1 — Table S1. The colorectal cancer screening participation rates in 2022 by the characteristics. Table S2. Factors related to colorectal cancer screening participation in 2022, among those who did not screen in 2021. [file CAM4-14-e70859-s001.docx]

Supplementary table 1 The Colorectal Cancer Screening Participation Rates in 2022 by the Characteristics

|  |  | Total | Non-Attendee on CRC Screening in 2021 | Attendee  on CRC Screening in 2021 | p-value |
| --- | --- | --- | --- | --- | --- |
|  |  | N=13,261 | N=7,894 | N=5,367 |  |
| Gender, n - % | Male | 6,535 (49.3%) | 3,811 (48.3%) | 2,724 (50.8%) | 0.005 |
|  | Female | 6,726 (50.7%) | 4,083 (51.7%) | 2,643 (49.2%) |  |
| Age, mean (SD) | | 57.16 (10.21) | 56.41 (10.31) | 58.28 (9.95) | <0.001 |
| Highest Education | Middle / High School | 3,959 (29.9%) | 2,478 (31.4%) | 1,481 (27.6%) | <0.001 |
|  | Vocational School/Junior College/Technical College | 3,268 (24.6%) | 2,023 (25.6%) | 1,245 (23.2%) |  |
|  | Universityand above | 6,034 (45.5%) | 3,393 (43.0%) | 2,641 (49.2%) |  |
| Occupation | Employer | 473 (3.6%) | 288 (3.6%) | 185 (3.4%) | <0.001 |
|  | Self-employed | 746 (5.6%) | 529 (6.7%) | 217 (4.0%) |  |
|  | Employee | 7,050 (53.2%) | 3,961 (50.2%) | 3,089 (57.6%) |  |
|  | Unemployed | 4,992 (37.6%) | 3,116 (39.5%) | 1,876 (35.0%) |  |
| Annual Household Income | 3 million or less | 2,306 (17.4%) | 1,523 (19.3%) | 783 (14.6%) | <0.001 |
|  | Greater than 3 million Yen | 8,141 (61.4%) | 4,598 (58.2%) | 3,543 (66.0%) |  |
|  | No response | 2,814 (21.2%) | 1,773 (22.5%) | 1,041 (19.4%) |  |
| Alcohol Use | Never | 4,977 (37.5%) | 3,103 (39.3%) | 1,874 (34.9%) | <0.001 |
|  | Ever | 902 (6.8%) | 596 (7.6%) | 306 (5.7%) |  |
|  | Current | 7,382 (55.7%) | 4,195 (53.1%) | 3,187 (59.4%) |  |
| Comorbidity | Absent | 9,022 (68.0%) | 5,521 (69.9%) | 3,501 (65.2%) | <0.001 |
|  | Present | 4,239 (32.0%) | 2,373 (30.1%) | 1,866 (34.8%) |  |
| State of emergency in 2021 for the place of residence | Not issued | 3,092 (23.3%) | 1,757 (22.3%) | 1,335 (24.9%) | <0.001 |
|  | Issued | 10,169 (76.7%) | 6,137 (77.7%) | 4,032 (75.1%) |  |
| Fear of Covid-19 scale score | Less than 21 | 8,579 (64.7%) | 5,024 (63.6%) | 3,555 (66.2%) | 0.002 |
|  | 21 and Greater | 4,682 (35.3%) | 2,870 (36.4%) | 1,812 (33.8%) |  |
| Colorectal cancer screening attendance before COVID-19 pandemic | Regular screening participation | 5,033 (38.0%) | 752 (9.5%) | 4,281 (79.8%) | <0.001 |
|  | Irregular screening participation | 2,604 (19.6%) | 1,878 (23.8%) | 726 (13.5%) |  |
|  | Never participation | 5,624 (42.4%) | 5,264 (66.7%) | 360 (6.7%) |  |

Supplementary table2 Factors Related to Colorectal Cancer Screening Participation in 2022, among Those Who did not Screen in 2021

|  |  | Non-attendee on CRC screening in 2021 | | | | | | | |
| --- | --- | --- | --- | --- | --- | --- | --- | --- | --- |
|  |  |  |  |  |  |  |  |  |  |
|  |  | Other reason  Willing to CRC screening | | COVID-19 Related reason  Willing to CRC screening | | Other Reason  Unwilling to CRC screening | | COVID-19 Related Reason  Unwilling to Participate | |
|  |  |  |  |  |  |  |  |  |  |
|  |  | aIRR (95% CI) | P | aIRR (95% CI) | P | aIRR (95% CI) | P | aIRR (95% CI) | P |
| Gender | Female | Reference |  | Reference |  | Reference |  | Reference |  |
|  | Male | 1.15 (0.95-1.38) | 0.164 | 1.31 (1.02-1.68) | 0.034 | 1.41 (1.12-1.78) | 0.004 | 1.42 (1.16-1.73) | 0.001 |
| Age | 70yr | Reference |  | Reference |  | Reference |  | Reference |  |
|  | 40yr | 0.68 (0.51-0.89) | 0.006 | 0.95 (0.67-1.33) | 0.742 | 1.36 (0.94-1.97) | 0.099 | 0.92 (0.65-1.29) | 0.616 |
|  | 50yr | 0.66 (0.50-0.86) | 0.003 | 1.01 (0.73-1.40) | 0.939 | 1.01 (0.69-1.46) | 0.976 | 0.90 (0.64-1.27) | 0.546 |
|  | 60yr | 0.99 (0.78-1.25) | 0.936 | 1.02 (0.77-1.36) | 0.885 | 1.07 (0.75-1.52) | 0.700 | 0.97 (0.70-1.35) | 0.853 |
| Marital Status | Married | Reference |  | Reference |  | Reference |  | Reference |  |
|  | Never married | 0.90 (0.72-1.13) | 0.374 | 0.77 (0.57-1.03) | 0.082 | 0.63 (0.48-0.83) | 0.001 | 0.82 (0.66-1.02) | 0.078 |
|  | Widowed | 0.90 (0.59-1.37) | 0.625 | 1.12 (0.66-1.88) | 0.679 | 1.05 (0.57-1.91) | 0.886 | 0.96 (0.56-1.67) | 0.895 |
|  | Divorced | 1.11 (0.84-1.47) | 0.47 | 0.96 (0.64-1.42) | 0.820 | 0.87 (0.61-1.24) | 0.439 | 1.07 (0.81-1.42) | 0.623 |
| Highest Education | University and above | Reference |  | Reference |  | Reference |  | Reference |  |
|  | Middle / High School | 0.89 (0.74-1.08) | 0.255 | 1.03 (0.81-1.30) | 0.819 | 0.96 (0.76-1.21) | 0.719 | 1.04 (0.85-1.26) | 0.732 |
|  | Vocational School/Junior College/Technical College | 0.91 (0.74-1.12) | 0.389 | 0.95 (0.73-1.23) | 0.676 | 1.00 (0.78-1.29) | 0.998 | 0.94 (0.75-1.18) | 0.619 |
| Employment Type | Employee | Reference |  | Reference |  | Reference |  | Reference |  |
|  | Employer | 1.06 (0.74-1.52) | 0.747 | 0.56 (0.31-0.98) | 0.042 | 1.24 (0.82-1.87) | 0.302 | 0.99 (0.66-1.46) | 0.945 |
|  | Self-employed | 0.66 (0.46-0.95) | 0.023 | 0.69 (0.45-1.06) | 0.088 | 0.72 (0.46-1.11) | 0.135 | 0.50 (0.34-0.75) | 0.001 |
|  | Unemployed | 0.75 (0.62-0.91) | 0.003 | 0.86 (0.69-1.09) | 0.213 | 0.55 (0.43-0.72) | p<0.001 | 0.58 (0.46-0.73) | p<0.001 |
| Annual Household Income | Greater than 3 Million Yen | Reference |  | Reference |  | Reference |  | Reference |  |
|  | Less than 3 Million Yen | 0.90 (0.72-1.14) | 0.391 | 1.02 (0.77-1.35) | 0.889 | 0.77 (0.57-1.05) | 0.100 | 0.88 (0.69-1.12) | 0.299 |
|  | No response | 1.00 (0.81-1.22) | 0.959 | 1.01 (0.79-1.29) | 0.930 | 0.92 (0.72-1.19) | 0.521 | 0.85 (0.69-1.06) | 0.150 |
| State of emergency in 2021 for the place of residence | Not issued | Reference |  | Reference |  | Reference |  | Reference |  |
|  | Issued | 0.93 (0.78-1.11) | 0.405 | 1.09 (0.86-1.38) | 0.488 | 1.13 (0.89-1.42) | 0.317 | 0.99 (0.81-1.21) | 0.910 |
| Fear of Covid-19 scale score | Less than 21 | Reference |  | Reference |  | Reference |  | Reference |  |
|  | 21 and Greater | 1.17 (1.00-1.38) | 0.051 | 0.97 (0.79-1.17) | 0.716 | 1.49 (1.23-1.81) | <0.001 | 1.26 (1.07-1.49) | 0.006 |
| Smoking | Never-user | Reference |  | Reference |  | Reference |  | Reference |  |
|  | Former-user | 0.94 (0.77-1.15) | 0.528 | 1.01 (0.79-1.30) | 0.919 | 1.08 (0.85-1.37) | 0.547 | 0.92 (0.73-1.16) | 0.458 |
|  | Current Smoking (paper) | 0.89 (0.68-1.16) | 0.394 | 0.93 (0.68-1.28) | 0.656 | 0.86 (0.62-1.20) | 0.377 | 0.86 (0.66-1.12) | 0.266 |
|  | Current Smoking (electronic) | 0.94 (0.64-1.39) | 0.771 | 0.96 (0.62-1.48) | 0.853 | 1.02 (0.68-1.54) | 0.913 | 1.06 (0.76-1.48) | 0.715 |
|  | Current Smoking (both) | 0.98 (0.69-1.38) | 0.894 | 0.94 (0.59-1.52) | 0.814 | 0.92 (0.59-1.43) | 0.703 | 1.00 (0.70-1.41) | 0.979 |
| Alcohol Use | Never | Reference |  | Reference |  | Reference |  | Reference |  |
|  | Ever | 0.84 (0.61-1.18) | 0.317 | 0.88 (0.60-1.30) | 0.525 | 1.31 (0.93-1.84) | 0.126 | 0.78 (0.54-1.12) | 0.179 |
|  | Current | 1.01 (0.85-1.20) | 0.930 | 1.01 (0.81-1.25) | 0.940 | 1.11 (0.90-1.38) | 0.320 | 0.97 (0.81-1.16) | 0.742 |
| Comorbidities | Present | Reference |  | Reference |  | Reference |  | Reference |  |
|  | Absent | 0.96 (0.81-1.14) | 0.649 | 0.87 (0.71-1.07) | 0.194 | 0.84 (0.68-1.04) | 0.105 | 0.94 (0.77-1.14) | 0.521 |

aIRR: adjusted incidence risk ratio
